# High Conversion Efficiency in Intrinsic High Power‐Density Mg_2_Sn‐GeTe Thermoelectric Generator

**DOI:** 10.1002/advs.202506997

**Published:** 2025-07-30

**Authors:** Xinzhi Wu, Longquan Wang, Airan Li, Gang Wu, Zhao Hu, Fei Frank Yun, Takao Mori

**Affiliations:** ^1^ Research Center for Materials Nanoarchitectonics (MANA) National Institute for Materials Science (NIMS) Tsukuba 305–0044 Japan; ^2^ Graduate School of Pure and Applied Science University of Tsukuba 1‐1‐1 Tennodai Tsukuba Ibaraki 305–8671 Japan

**Keywords:** conversion efficiency, Mg2Sn‐GeTe device, power density, thermoelectric generators

## Abstract

Thermoelectric generators (TEGs) offer a sustainable solution for thermal energy harvesting, where maximizing energy output necessitates achieving both high power density and high conversion efficiency. However, TEGs with intrinsically high power density by employing high power factor materials often face efficiency limitations due to their usually high thermal conductivity. Here, intrinsically high power density TEGs based on Mg_2_Sn‐GeTe for the first time is presented, simultaneously delivering a remarkable conversion efficiency of 9% under a temperature gradient of 418 K, thereby setting a new benchmark in the field. This exceptional performance is attributed to the significant balance between the moderating carrier and phonon transport in Mg_2_Sn, enabled by a stepwise aliovalent Sb and Bi solid solution, without over‐compromising its outstanding power factor. Consequently, a high thermoelectric figure of merit of 1.4 is achieved in Mg_2_Sn_0.8_(Sb_0.5_Bi_0.5_)_0.2_. The high‐performance Mg_2_Sn–GeTe TEGs introduced here represent a significant advancement in thermoelectric technology, offering an innovative and efficient solution for off‐grid energy supply in waste‐heat‐rich environments.

## Graphical abstract

First Mg_2_Sn–GeTe thermoelectric generator achieves 9% efficiency by strategically modulating power factor–thermal conductivity interplay in intrinsically high‐power‐density system.

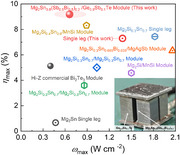

## 1 Introduction

Industrial processes are a major source of waste heat, with up to 60% of energy often lost as residual heat, exacerbating environmental concerns.^[^ ^1^ ^]^ Coupled with the ongoing global energy crisis, the increasing environmental challenges, and the urgent push toward carbon neutrality and peak emissions targets, there is an escalating demand for sustainable energy conversion technologies.^[^ ^2^, ^3^ ^]^ Thermoelectric generators (TEGs) present a promising solution, as they directly convert heat into electricity without producing emissions or noise, offering a clean and efficient method for energy recovery.^[^ ^4^, ^5^ ^]^ This is especially relevant in scenarios with abundant heat sources, where TEGs can promisingly address energy and environmental challenges.^[^ ^4^ ^]^

To expand their applicability across diverse heat‐rich environments, enhancing the energy output of TEGs is crucial, as it directly determines their capability to meet varied power demands.^[^ ^6^ ^]^ Maximizing the energy output of TEGs requires both high power density and high conversion efficiency. Power density ensures the amount of energy generated per unit area, while conversion efficiency dictates how effectively heat is converted into electrical energy and minimizes energy losses. Power density is primarily influenced by the power factor of thermoelectric (TE) materials, while conversion efficiency is determined by the thermoelectric figure of merit (ZT) without considering device size and interfaces.^[^ ^7^ ^]^ Interfacial resistance has long posed a bottleneck for device advancement,^[^ ^8^ ^]^ and encouragingly, recent advances have significantly mitigated this issue, reducing interfacial resistivity to as low as 10 µΩ·cm^2^,^[^ ^9^, ^10^, ^11^, ^12^ ^]^ which limits *ZT* losses below 5%.^[^ ^13^ ^]^ TEGs with high power density hold unique advantages in practical application for waste heat recovery, such as in industrial environments or automotive exhaust heat recovery.^[^ ^14^ ^]^ Choosing TE materials with a high power factor can facilitate the development of high‐power‐density TEGs. However, the usually high thermal conductivity of such TE materials often leads to a reduced *ZT*, ultimately lowering the conversion efficiency of these TEGs. For instance, some typical materials NiAu, Fe_2_VAl, NbFeSb, Mg_2_Sn, etc.^[^ ^15^, ^16^, ^17^ ^]^ Therefore, balancing the trade‐off between the electrical power factor and thermal conductivity via moderating carrier and phono transport remains a significant challenge in developing TEGs with high power density and conversion efficiency.

The n‐type Mg_2_Sn‐based^[^ ^18^, ^19^, ^20^, ^21^, ^22^, ^23^, ^24^, ^25^, ^26^, ^27^ ^]^ and p‐type GeTe‐based^[^ ^28^, ^29^, ^30^, ^31^ ^]^ compounds stand out as promising candidates for power‐oriented TEGs due to their high power factor, which grants them intrinsic advantages in achieving high power density in mid‐temperature applications. Additionally, their environmentally friendly composition makes them sustainable options. The p‐type GeTe has garnered significant attention for its high power factor and further reduced low thermal conductivity by entropy, defect or charge transfer engineering, with numerous reports demonstrating a *ZT* value exceeding 2.^[^ ^32^, ^33^, ^34^, ^35^, ^36^ ^]^ However, the progress in enhancing the *ZT* of Mg_2_Sn has lagged behind that of GeTe. Zaitsev et al.^[^ ^18^ ^]^ first reported a *ZT* of 1.1 at 723 K in 2006, with subsequent enhancements to *ZT* 1.4 and a power factor of 5 W m^−1^ K^−2^.^[^ ^20^, ^37^ ^]^ Since then, interest in Mg_2_Sn has surged, culminating in recent breakthroughs,^[^ ^19^, ^20^, ^24^, ^38^, ^39^ ^]^ achieving *ZT* 1.6 for Mg_2_(Si_0.4_Sn_0.6_)_0.985_Sb_0.015_ at 823 K.^[^ ^40^ ^]^ However, experimental validation of these high‐*ZT* materials at the device level remains unreported. Despite its inherently high power factor, the Mg_2_Sn exhibits a thermal conductivity of over 5 W m^−1^ K^−1^ ≈300 K, which imposes a fundamental constraint on the conversion efficiency of fabricated TEGs. Nevertheless, addressing the intrinsic high thermal conductivity of n‐type Mg_2_Sn while maintaining high power density remains an overlooked challenge at the device level. To date, no practical implementation of p‐n TEGs integrating these two high‐power‐factor TE materials has been reported.

In this study, we present a novel Mg_2_Sn─GeTe TEGs with the intrinsic high‐power‐density, achieving an impressive conversion efficiency of 9% under a temperature gradient of 418 K. This performance is realized through a stepwise aliovalent Sb and Bi solid solution strategy on Mg_2_Sn, which effectively balances the power factor and thermal conductivity via moderating carrier and phono transport, achieving a low lattice thermal conductivity of 1.8 W·m^−1^·K^−1^ and a peak *ZT* of 1.4. Notably, this optimization entails only a ≈20% reduction in power factor, yet yields a ∼47% decrease in lattice thermal conductivity—an outcome that substantially improves *ZT* and offers a promising route toward an intrinsic high‐power‐density system. These advancements address the inherent trade‐off between electrical properties and thermal conductivity, culminating in a high‐performance Mg_2_Sn_0.8_(Sb_0.5_Bi_0.5_)_0.2_–Ge_0.9_Sb_0.1_Te TEG. Our optimized TEGs provide a sustainable and efficient solution for energy harvesting in waste‐heat‐rich, mid‐temperature environments, offering a practical reference for other intrinsic high‐power density TE devices.

## 2 Main Text

### 2.1 Stepwise Optimized Materials and Device Performance

**Figure 1:**
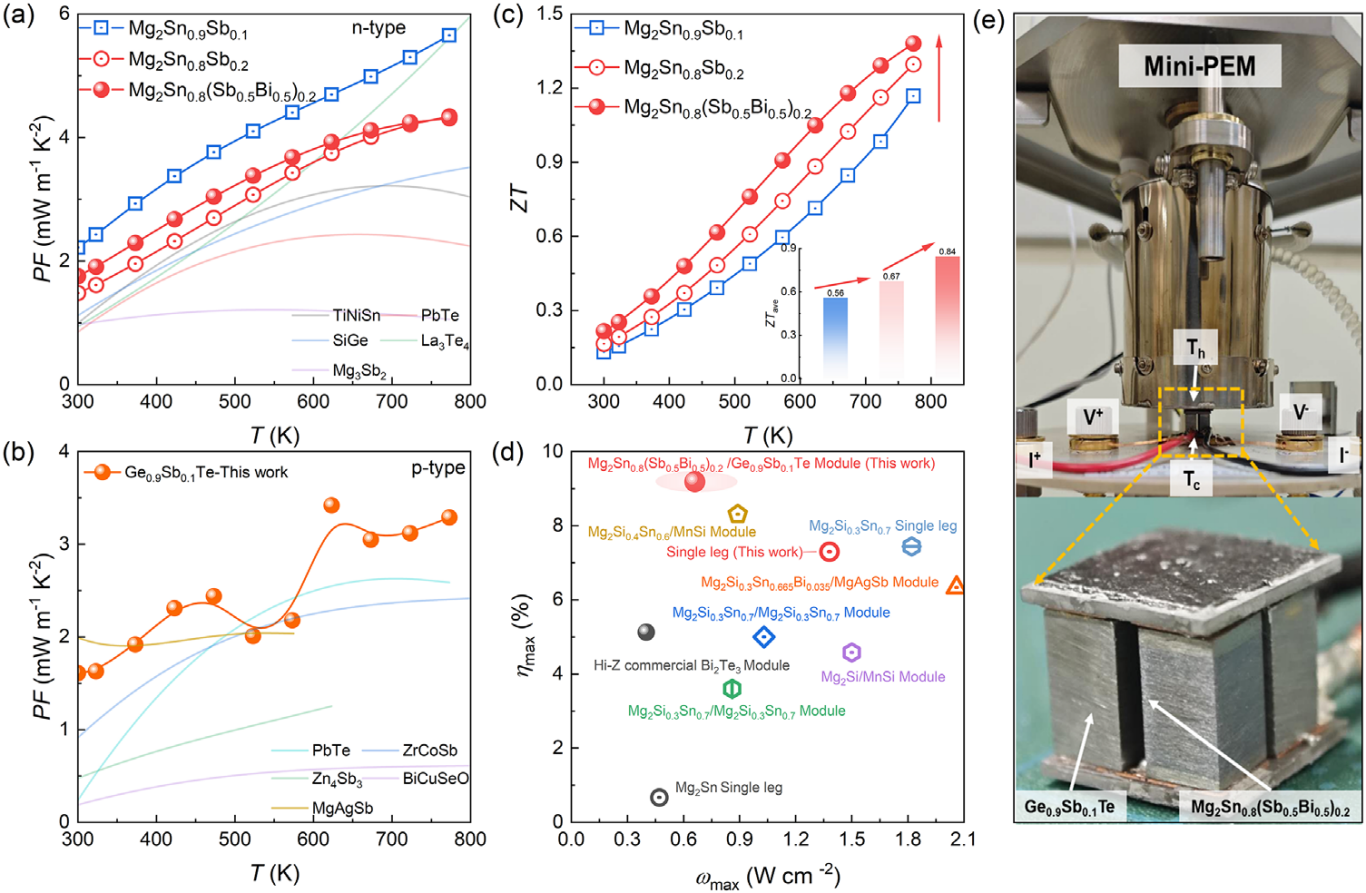
Stepwise Optimized performance of TE materials and device via aliovalent solid solution Strategy. The temperature‐dependent power factor variations for n‐type Mg_2_Sn a) and p‐type GeTe b), compared to several benchmark TE materials.^[^ ^41^ ^]^ c) *ZT* values as a function of temperature, and the inset shows the *ZT*_ave_ values over the temperature range from room temperature to 773 K. d) A 2D map correlating the maximum power density (*ω*_max_) with the maximum conversion efficiency (*η*_max_) for Mg_2_Sn‐based TE devices, along with comparative data from the literature.^[^ ^42^, ^43^, ^44^, ^45^, ^46^, ^47^ ^]^ e) Photograph of the device setup in the commercial instrument Mini‐PEM, with a close‐up view of the device configuration.

Drawing inspiration from the intrinsic advantages of high power, we demonstrate a strategy that effectively balances the power factor and thermal conductivity through aliovalent solid solutions Sb and Bi in Mg_2_Sn‐based TE materials, leading to significantly improved *ZT*. As shown in **Figure** **1**a, the power factor of Mg_2_Sn_0.9_Sb_0.1_ reaches ≈7 mW m^−1^ K^−2^ at 773 K. Under similar Sb doping concentration, the power factor of Ge_0.9_Sb_0.1_Te with high *ZT* exceeds ≈3 mW m^−1^ K^−2^ at 773 K, also surpassing most conventional p‐type TE materials^[^ ^41^ ^]^ such as PbTe, ZrCoSb, Zn_4_Sb_3_, BiCuSeO, and MgAgSb^[^ ^48^ ^]^ (Figure 1b), thereby providing a fundamental requirement for high‐power‐density devices. However, the *ZT* of Mg_2_Sn‐based TE materials with an average *ZT_ave_* of 0.56 for Mg_2_Sn_0.9_Sb_0.1_ remains lower than that of GeTe (Figure S1, Supporting Information), As shown in Figure 1c, increasing the Sb doping concentration raises the *ZT_ave_* from 0.56 for Mg_2_Sn_0.9_Sb_0.1_ to 0.67 for Mg_2_Sn_0.8_Sb_0.2_. Similarly, the peak power factor decreases from over 7 mW m^−1^ K^−2^ to ≈5 mW m^−1^ K^−2^ as Sb doping concentration increases from 0.1 to 0.2. Nevertheless, at Sb = 0.2, the peak power factor remains higher than that of other n‐type TE materials^[^ ^41^ ^]^ such as TiNiSn, SiGe, PbTe, La_3_Te_4_, and Mg_3_Sb_2_, making it an ideal baseline for further optimizing its thermal conductivity (Figure 1a).

The addition of Bi raises the average *ZT_ave_* to 0.84 in Mg_2_Sn_0.8_(Sb_0.5_Bi_0.5_)_0.2_ (Figure 1c), even with a slight improvement in the power factor compared with the Mg_2_Sn_0.8_Sb_0.2_ (Figure 1a), particularly between room temperature to 773 K temperature range. The *ZT* of the repeated batches exhibits fluctuations within 5%, confirming the reliability of our findings (Figure S2, Supporting Information). The assembled 2 pair Mg_2_Sn_0.8_(Sb_0.5_Bi_0.5_)_0.2_‐Ge_0.9_Sb_0.1_Te device and the testing setup are shown in Figure 1d,e, demonstrating a power density of 0.7 W cm^−2^ and conversion efficiency of 9%. It is worth noting that the power density is also affected by the device's physical dimensions, suggesting that further improvements could be achieved through structural optimization of the TE device. In addition, the integration with p‐type Ge_0.9_Sb_0.1_Te results in significantly enhanced conversion efficiency, which is higher than that of other Mg_2_Sn‐based TEGs such as Mg_2_Si_0.3_Sn_0.7_‐Mg_2_Si_0.3_Sn_0.7_,^[^ ^43^, ^46^ ^]^ Mg_2_Si‐MnSi,^[^ ^42^ ^]^ and Mg_2_Si_0.3_Sn_0.7_‐MgAgSb TEGs.^[^ ^47^ ^]^

### 2.2 TE Performance

Sb and Bi play a pivotal role in optimizing the *ZT* of Mg_2_Sn, necessitating a detailed investigation of their individual effects. In this section, we elucidate the underlying mechanisms of Sb and Bi aliovalent solid solution, specifically the Bi‐induced retention of power factor and the Sb/Bi‐driven reduction in thermal conductivity. This analysis highlights the delicate balance between moderating carrier transport to mitigate power factor degradation and enhancing phonon scattering to suppress thermal conductivity.

**Figure 2:**
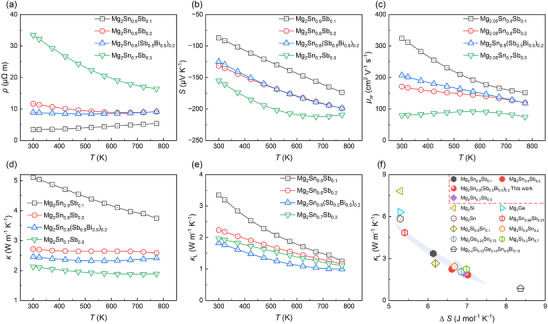
TE properties of Mg_2_Sn‐based materials. a–c) Temperature dependence of electrical resistivity (*ρ*), Seebeck coefficient (*S*), and weighted mobility (*µ*_w_) for various compositions. (e) The temperature‐dependent thermal conductivity *κ*, and lattice *κ*_L_. f) The relationship between *κ*_L_ and configuration entropy (Δ*S*), along with comparative data from the literature.^[^ ^23^ ^]^

XRD and SEM analyses confirm the phase purity and compositional uniformity of the Sb‐ and Bi‐doped samples (Figures S3–S6, Supporting Information), consistent with the high solubility of these elements in the Mg_2_Sn matrix.^[^ ^23^, ^49^, ^50^ ^]^ **Figure** **2**a–d illustrates the electrical transport properties of the Mg_2_Sn aliovalent element solid solution system. As shown in Figure 2a, the temperature‐dependent electrical resistivity (*ρ*) curves reveal a distinct transition in conduction behavior. At Sn = 0.9, the system exhibits degenerate semiconductor behavior, characterized by a decreasing *ρ* with increasing temperature, indicative of metallic conduction. In contrast, as the Sn content decreases to 0.7, the material transitions to non‐degenerate semiconductor behavior, with *ρ* showing a stronger temperature dependence, characteristic of thermally activated transport. Figure 2b presents the Seebeck coefficient (*S*), which increases monotonically with temperature for all compositions. Notably, the *S* also shows a general increase with higher Sb content. However, the power factor decreases systematically with decreasing Sn content, indicating that lower Sn concentrations adversely affect the overall electrical performance (Figure 1a). This trend suggests that electrical properties are strongly influenced by the Sn and Sb/Bi content. Sb doping induces a non‐monotonic evolution in carrier effective mass, extracted from Pisarenko analysis: effective mass increases from ≈2.9 *m*_e_ to ≈3.4 *m*_e_ at Sb = 0.2, then slightly decreases to ≈3.1 *m*_e_ at Sb = 0.3 (Figures S7, Supporting Information). This mirrors trends in Bi‐doped Mg_2_Sn, likely attributed to band structure changes driven by heavy and light band convergence.^[^ ^49^ ^]^ To elucidate the mechanisms underlying these observations, the weighted mobility (*µ*_w_) was analyzed using Snyder's modified model:^[^ ^51^ ^]^

(1) $$\begin{array}{ccc} \mu_{w} & = & \frac{3h^{3}\sigma }{8\pi e{\left(2m_{e}k_{B}T\right)}^{3/2}}\left[\frac{\exp\left[\frac{\left|S\right|}{k_{B}/e}-2\right]}{1+\exp\left[-5\left(\frac{\left|S\right|}{k_{B}/e}-1\right)\right]}\right.\\ & & \left.+\frac{\frac{3}{\pi^{2}}\frac{\left|S\right|}{k_{B}/e}}{1+\exp\left[5\left(\frac{\left|S\right|}{k_{B}/e}-1\right)\right]}\right] \end{array}$$

Where *σ* is experimental measurements of electrical conductivity, *k*_B_ is the Boltzmann constant, *h* is the Planck constant, *e* is the electron charge, and *m*_e_ is the free electron mass. The *µ*_w_ exhibits significant variations, particularly around room temperature, where it decreases as the Sn content decreases (Figure 2c). This trend suggests that *µ*_w_ could be a key factor influencing *ρ*, aligning with grain boundary scattering mechanisms commonly observed in Mg_2_Si, Mg_3_Sb_2,_ and half‐Heusler TE materials.^[^ ^52^, ^53^ ^]^ The *µ*_w_ is related to the *µ*_H_ and the m^*^ by the relation *µ*_w_ ∝ *µ*_H_ /(m^*^/*m_e_*)*^3/2^*.^[^ ^51^ ^]^ Across the Mg_2_Sn_1‐_ *_x_* Sb*_x_* series, the *m* ^*^ exhibits a slight variation (2.9‐3.4 *m*_e_), whereas *µ*_H_ decreases sharply from 43.7 to 11.1 4 cm^2^ V^−1^ s^−1^ as Sb content increases from *x*  =  0.1 to 0.3, consistent with the corresponding drop in *µ*_w_ from 324.7 to 79.4 4 cm^2^ V^−1^ s^−1^ (FiguresS 8). It should be noted that *µ*_H_ is also influenced by the band structure of the material. According to the relationship *µ*_H_ ∝ *τ*/m^*^, the reduction in *µ*_H_ arises from the combined effects of changed effective mass and carrier scattering. In other words, the varied band structure with increasing Sb content affects the *µ*_H_. In addition, enhanced carrier scattering at higher Sb concentrations also contributes to decreased carrier mobility in Mg_2_Sn as the Sb content increases.

Figure 2d‐f presents the systematic variations in thermal properties. The thermal conductivity (*κ*) exhibits a decreasing trend with Sb increases (Figure 2d). A monotonic reduction in *κ* is observed with decreasing Sn content from Mg_2_Sn_0.9_Sb_0.1_ to Mg_2_Sn_0.8_Sb_0.7_. For example, the *κ* decreases from 5.1 W m^−1^ K^−1^ for Mg_2_Sn_0.9_Sb_0.1_ to 2.1 W m^−1^ K^−1^ for Mg_2_Sn_0.7_Sb_0.3_ at room temperature. In the Mg_2_Sn_0.8_(Sb_0.5_Bi_0.5_)_0.2_, room‐temperature *κ* is ≈2.4 W m^−1^ K^−1^, which is slight lower than the value of Mg_2_Sn_0.8_Bi_0.2_ (2.7 W·m^−1^ K^−1^). The lattice conductivity (*κ*_L_) is plotted after subtracting the electronic contribution. With increasing Sb content, *κ*_L_ deviates from the *T* ^−1^ temperature dependence typical of phonon‐phonon scattering, indicating the dominance of strong phonon scattering from point defects and grain boundaries introduced by Sb/Bi solid solution (Figure 2e). Sound velocity measurements support these observations, showing a clear reduction with increasing Sb/Bi content. The sound velocity decreases from 3005 m s^−1^ for Mg_2_Sn_0.9_Sb_0.1_ to 2852 m s^−1^ for Mg_2_Sn_0.8_(Sb_0.5_Bi_0.5_)_0.2_, accompanied by a progressive reduction in *κ*_L_, which declines from 3.4 W m^−1^ K^−1^ for Mg_2_Sn_0.8_Sb_0.2_ to 1.8 W m^−1^ K^−1^ for Mg_2_Sn_0.8_(Sb_0.5_Bi_0.5_)_0.2_ (Figures S9, Supporting Information). Debye–Callaway analysis reveals that the dominant contribution to the reduced *κ*_L_ stems from point defect scattering, consistent with behaviors observed in Bi‐containing Mg_2_Sn‐based TE materials. The *κ*_L_ of our samples is slightly higher than that of the Bi‐containing counterparts, likely due to weaker point‐defect scattering. This is supported by the calculated mass fluctuation parameters, where Γ = 0.0445, for Mg_2_Sn_0.8_(Sb_0.5_Bi_0.5_)_0.2_—lower than Γ = 0.0697 for Mg_2_Sn_0.8_Bi_0.2_ (Figures S10, Supporting Information). In addition, the reduction in *κ*_L_ can be attributed to the disorder induced by increased entropy, which serves as a promising strategy for optimizing TE performance.^[^ ^54^, ^55^, ^56^ ^]^ Figure 2f illustrates the significant reduction in *κ*_L_ with increasing configurational entropy (Δ*S*). As Δ*S* increases from 5.3 to 7.0 J mol^−1^ K^−1^, the *κ*_L_ decreases from 5.8 W m^−1^ K^−1^ in Mg_2_Sn to 1.8 W m^−1^ K^−1^ in Mg_2_Sn_0.8_(Sb_0.5_Bi_0.5_)_0.2_. To further enhance the entropy could effectively reduce the *κ*_L_ to ≈0.8 W m^−1^ K^−1^.^[^ ^23^ ^]^ However, this study lies in achieving the optimum compromise of electrical and thermal transport, and the optimized *ZT* achieved in this work is 1.4, which is higher than the 1.3 reported for high‐entropy systems in previous studies.^[^ ^23^ ^]^ In addition, this trend also aligns with lattice softening and enhanced phonon scattering due to Bi doping,^[^ ^57^ ^]^ which is more pronounced than that observed with Sb doping^[^ ^40^ ^]^ in Mg_2_Sn‐based TE materials. Introducing chemical complexity and disorder by Bi can significantly influence lattice dynamics, electronic structure, and thermal conductivity. Specifically, Bi has a much larger atomic mass (208.98 u) compared to Sb (121.76 u), leading to a significant increase in the mass fluctuation parameter.^[^ ^24^, ^58^ ^]^ Moreover, the atomic radius of Bi (160 pm) is notably larger than that of Sb (140 pm), which increases the strain fluctuation parameter through greater lattice mismatch, further amplifying phonon scattering.^[^ ^57^ ^]^

These combined effects result in a pronounced reduction in *κ*_L_ while preserving favorable electrical properties, ensuring a favorable trade‐off between thermal and electrical transport. The quality factor *B* is widely used to assess the combined influence of electron and phonon transport, highlighting the compositional optimization for peak thermoelectric performance:^[^ ^51^ ^]^

(2) $$B={\left(\frac{k_{B}}{e}\right)}^{2}\frac{8\pi e{\left(2m_{e}k_{B}T\right)}^{3/2}}{3h^{3}}\frac{\mu_{w}}{\kappa_{L}}T$$

Here, we also use this parameter B to quantify variations in material performance. The highest *B* value of 0.9 is observed in Mg_2_Sn_0.8_(Sb_0.5_Bi_0.5_)_0.2_ which is higher than the value of 0.7 in Mg_2_Sn_0.8_Sb_0.2_ (Figure S11, Supporting Information), resulting in a maximum *ZT* of 1.4 for Mg_2_Sn_0.8_(Sb_0.5_Bi_0.5_)_0.2_ (Figure 1c).

### 2.3 Interface Performance

**Figure 3:**
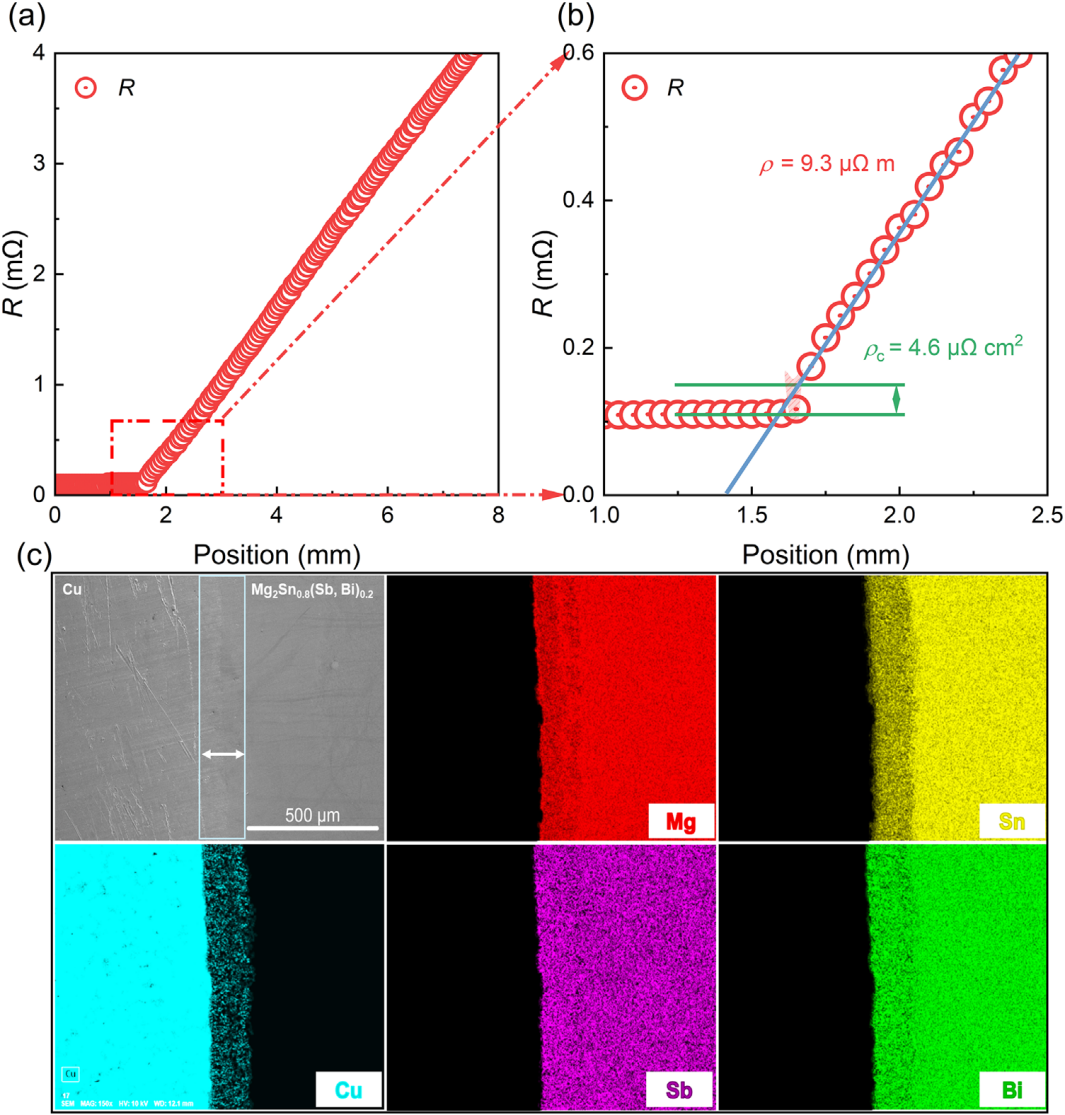
Electrical and microstructural characteristics of the Mg_2_Sn_0.8_(Sb_0.5_Bi_0.5_)_0.2_‐based material and electrode interface. a) Resistance *R* versus position curve, with the inset showing an optical photograph of the single‐leg device. b) Magnified view of the interface region in (a), highlighting the interface resistance determined from the resistance jump across the probe displacement. c) Scanning electron microscopy (SEM) image and corresponding energy dispersive spectroscopy (EDS) elemental maps of the interface region depicted in (a).

To validate the exceptional performance of the Mg_2_Sn_0.8_(Sb_0.5_Bi_0.5_)_0.2_ TE material, the TE device was fabricated using Cu as the electrode material. Note that Cu may not be optimal in terms of interfacial robustness; it provides a practical adopted platform for evaluating the optimized Mg_2_Sn‐based TE materials.^[^ ^43^, ^59^, ^60^ ^]^ **Figure** **3**a shows the characterization of the interface resistance of the n‐type single‐leg. After polishing the interface region, the resistance‐displacement curve was used to calculate the interface resistivity (*ρ*_c_). Notably, the transition region of the interface shows no significant resistance jumps, indicating acceptable electrical contact. A closer examination of the interface region in Figure 3b reveals a low *ρ*_c_ of 4.6 µΩ cm^2^. Additionally, the *ρ* of the Mg_2_Sn_0.8_(Sb_0.5_Bi_0.5_)_0.2_ was determined to be 9.3 µΩ m using a scanning probe method, which closely matches the value of 8.9 µΩ m obtained from ZEM measurements. This consistency demonstrates the reliability of the measurement system and confirms that the TE material retains its optimized electrical properties even after contact fabrication.

Microstructural analysis of the interface region was performed to further elucidate the underlying mechanisms. As shown in Figure 3c, the SEM image highlights a well‐bonded interface without noticeable voids or cracks, confirming the high‐quality fabrication process. However, EDS elemental mapping reveals an obvious interdiffusion of elements, with a diffusion length of ≈100 µm. This level of interdiffusion is consistent with prior reports using Cu as a thermoelectric interface material.^[^ ^61^ ^]^ The Cu is considered beneficial to its beneficial to n‐type transport behavior,^[^ ^62^ ^]^ which would not excessively deteriorate the n‐type TE performance shortly. Thus, this diffusion has limited effects on both the interface and the TE performance of the material over short durations.

According to Xiong et al.^[^ ^13^ ^]^ The device *ZT_D_* and material *ZT* can be quantitatively correlated by considering the *ρ* of TE material, *ρ*_c_, and device height *L*. They report that an *ρ*_c_ below 5 µΩ cm^2^ maintains more than 95% of the material *ZT*, for a 2 mm Bi_2_Te_3_ device.

(3) $$ZT_{D}=\frac{L}{\left(L+2\rho_{c}\sigma \right)}ZT$$

Therefore, the *ρ*_c_ reported in this work is well within this ideal range. Besides, the interface of p‐type Ti/Ge_0.9_Sb_0.1_Te also exhibits a low *ρ*_c_ of < 5 µΩ cm^2^ (Figures S12 and S13, Supporting Information). Note that this work aims to establish the consistency between the material and device and does not yet include the design or optimization of diffusion barriers for long‐term interfacial stability. Future studies could explore electrode interface stability, with further optimization through alloying strategies,^[^ ^61^ ^]^ phase diagram‐guided material selection,^[^ ^8^ ^]^ or self‐optimizing contact design.^[^ ^63^ ^]^

### 2.4 Device Performance

**Figure 4:**
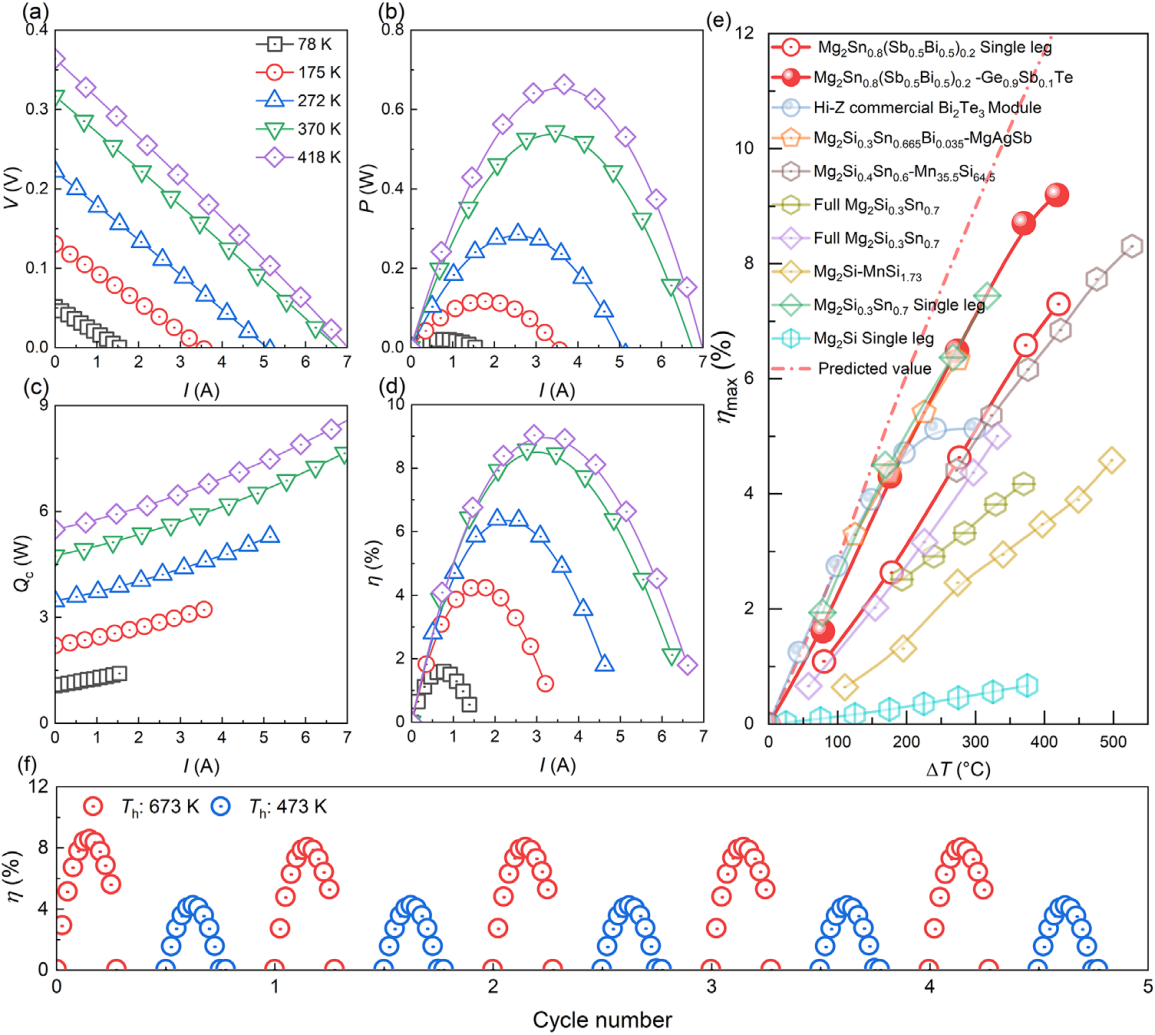
The power generation performance of the Mg_2_Sn_0.8_(Sb_0.5_Bi_0.5_)_0.2_‐Ge_0.9_Sb_0.1_Te module. Panels a–d) illustrate the Voltage 𝑉, Output power 𝑃, Heat flow at the cold side 𝑄𝑐, and Conversion efficiency 𝜂 as a function of current 𝐼 under various temperature differences Δ𝑇, respectively. e) Maximum conversion efficiency *η*_max_ as a function of temperature difference Δ*T*, compared to other reported Mg_2_Sn‐based TE devices,^[^ ^42^, ^43^, ^44^, ^45^, ^46^, ^47^, ^64^ ^]^ f) 𝜂 with the cycle number, *T*_h_ cycling at 473–673 K.

We paired n‐type Mg_2_Sn_0.8_(Sb_0.5_Bi_0.5_)_0.2_ with p‐type Ge_0.9_Sb_0.1_Te in this work for the first time and conducted power generation tests under various temperature gradients to validate the device's performance. The *V–I* characteristics exhibit linear discharge curves at various temperature differences (Δ*T*), with a negative slope indicative of power generation behavior (**Figure** **4**a). This linearity confirms good ohmic contact across the device under all operating conditions. The open‐circuit voltage increases from 0.05 V at Δ*T* = 78 K to 0.36 V at Δ*T* = 418 K. At the same Δ*T*, the output power initially increases with current, reaching a maximum when internal resistance equals the load resistance, and then decreases with further increases in current. As shown in Figure 4b, the maximum output power rises with increasing temperature difference, reaching 0.67 W at Δ*T* = 418 K. Similarly, the heat flow increases with current (Figure 4c), with contributions from both Joule heat and Fourier heat. The rise in open‐circuit heat flow across different Δ*T* is mainly attributed to Fourier heat transfer. Figure 4d illustrates the conversion efficiency (*η*) as a function of current, displaying a parabolic trend where *η* initially increases with current before declining. The *η*_max_ improves with increasing Δ*T*, reaching 9% under the Δ*T* of 418 K (Figure 4e). We also fabricated Mg_2_Sn_0.8_(Sb_0.5_Bi_0.5_)_0.2_ single‐leg devices for validation, and its *η*_max_ is 7.3% under the Δ*T* of 420 K (Figure S14, Supporting Information), slightly lower than the 7.5% reported by Chen et al.^[^ ^45^ ^]^ However, compared to other reported TE modules involving the n‐type Mg_2_Sn‐based with alternative p‐type TE materials,^[^ ^42^, ^43^, ^44^, ^45^, ^46^, ^47^, ^64^ ^]^ this combination achieves a high *η*_max_, underscoring its potential for practical TE applications. In addition, the single‐leg GeTe device demonstrated a conversion efficiency of 8.8% under a temperature gradient of 421 K (Figure S15, Supporting Information). Measured performance from the PEM mini system deviates from theoretical predictions (Figure S16, Supporting Information), likely due to parasitic line resistance during assembly and overestimated heat flux from radiative losses.^[^ ^65^ ^]^ Moreover, the device exhibits acceptable cycling stability, with no noticeable *η* degradation observed after five thermal cycles between 473 and 673 K (Figure 4f), as well as the output power (Figures S17, Supporting Information). Note that device performance is intrinsically governed by material TE properties, including power factor*, ZT*, as well as external factors such as device sized, *ρ*_c_, and temperature gradient. Herein, we focus on materials properties and demonstrate that balancing power factors and thermal conductivity via a stepwise Sb‐Bi aliovalent solid solution strategy enables high efficiency in intrinsically high power density thermoelectric modules.

## 3 Conclusion

This work presents a novel combination of Mg_2_Sn and GeTe, intrinsically high‐power‐density devices that have not been previously explored. Our design approach addresses the challenge of efficiency limitations due to their usually high thermal conductivity by incorporating aliovalent Sb and Bi solid solutions in Mg_2_Sn, which effectively reduces thermal conductivity while retaining the power factor. This results in a lattice thermal conductivity of 1.8 W m^−1^ K^−1^ and a peak *ZT* of 1.4 in Mg_2_Sn_0.8_(Sb_0.5_Bi_0.5_)_0.2_. The Mg_2_Sn‐GeTe module achieves a *η*_max_ of 9% at a high power density of 0.7 W·cm^−2^ under a 418 K temperature gradient, offering a promising strategy for efficient thermal energy conversion and advancing the development of sustainable energy solutions in intrinsically high‐power‐density TEG.

## 4 Experimental Section

### Materials Synthesis

The Mg_2.06_Sn_0.9_Sb_0.1_, Mg_2.06_Sn_0.8_Sb_0.2_, Mg_2.06_Sn_0.8_(Sb_0.5_Bi_0.5_)_0.2_, and Mg_2.06_Sn_0.7_Sb_0.3_ compounds were synthesized using high‐purity magnesium turnings (99.95%), tin shots (99.99%), antimony shots (99.999%), and bismuth shots (99.999%). Excess magnesium is denoted as Mg_2_ in the text, with similar abbreviations for other compositions. For example, Mg_2.06_Sn_0.9_Sb_0.1_ is denoted as Mg_2_Sn_0.9_Sb_0.1_. The raw materials were weighed according to their stoichiometric ratios and mechanically alloyed for 7 h using an SPEX‐8000D high‐energy mill under an argon atmosphere. The resulting powders were consolidated into bulk samples via vacuum spark plasma sintering at 873 K and 60 MPa for 5 min using the SPS‐1080 System (SPS SYNTEX INC). The Ge_0.9_Sb_0.1_Te samples, bulk Ge (99.99%), Te (99.99%), and Sb (99.99%) were weighed stoichiometrically and sealed in evacuated quartz tubes. The tubes were gradually heated to 1323 K, held at this temperature for 20 h to ensure complete mixing and reaction, and then cooled to room temperature. The alloys obtained were ground into fine powders using an agate mortar and consolidated by SPS under an axial pressure of 60 MPa at 873 K for 10 min (SPS‐322Lx, Dr. Sintering).

### Characterization and Measurements

The phase and phase transitions were investigated using a X‐ray diffractometer (Rigaku SmartLab 9 kW). The electrical transport properties, including *S* and the *σ*, were measured by ZEM‐3 (Advance Riko, ± 5% uncertainty), and the thermal transport property *κ* was calculated by the formula: *κ* = *DρC_p_*, where the *D* represents thermal diffusivity and was measured by LFA467 (Netzsch, ±3% uncertainty). The sample density *ρ* and *C_p_* were obtained by the Archimedes method and Dulong‐Petit law. The Hall measurements were performed using a Quantum Design Physical Property Measurement System. The magnetic field was swept from −4 T to +4 T at room temperature. The Hall coefficient *R*_H_ was extracted from the slope of the linear fit of Hall resistance versus magnetic field. The carrier concentration *n* and Hall mobility *µ_H_* were calculated using the relation *n* = 1/(*eR*_H_) and *µ* = *σR*_H_, where *e* is the elementary charge. The contact resistance of the TE junctions was measured by a two‐axis resistance distribution measurement instrument (S1331, Mottainai Energy). The thermoelectric modules were tested by the commercial instrument Mini‐PEM (Advance Riko).

### TE Device Fabrication and Simulation

The Mg_2_Sn_0.8_(Sb_0.5_Bi_0.5_)_0.2_ TE leg was fabricated by sandwiching two layers of Cu powders as interface materials, followed by SPS (SPS‐322Lx, Dr. Sintering) at 873 K and 60 MPa for 5 min. The sintered TE legs were then cut into dice with dimensions of ≈3.8 × 3.8 × 6.5 mm^3^. Two‐pair TE modules were constructed using these p‐type Ge_0.9_Sb_0.1_Te legs and n‐type Mg_2_Sn_0.8_(Sb_0.5_Bi_0.5_)_0.2_ TE legs. A Ti diffusion barrier layer was introduced at the GeTe side to suppress interfacial reactions. The dimensions of the two‐pair TE module are ≈10 × 10 × 10 mm^3^, with the AlN ceramic plate and copper electrode being 0.635 and 0.2 mm, respectively. Finite‐element simulations were performed using COMSOL Multiphysics to model both the single‐leg and two‐pair TE devices.

## Conflict of Interest

The authors declare no conflict of interest.

## Author Contributions

Xinzhi Wu wrote the original manuscript. Takao Mori designed the project. Xinzhi Wu, Longquan Wang, and Airan Li prepared the samples and carried out the measurements. Xinzhi Wu, Gang Wu, Zhao Hu, and Fei Frank Yun analyzed the data. Takao Mori supervised the whole project. All the authors discussed, reviewed, and edited the manuscript.

## Supporting information



Supporting Information

## Data Availability

The data that support the findings of this study are available from the corresponding author upon reasonable request.
