# Supplementary material for: High Conversion Efficiency in Intrinsic High Power‐Density Mg2Sn‐GeTe Thermoelectric Generator
Source: Adv Sci (Weinh). 2025 Jul 30;12(40):e06997. doi: 10.1002/advs.202506997 (PMC12561404; doi:10.1002/advs.202506997)
Supplement: Supplementary file 1 — Supporting Information [file ADVS-12-e06997-s001.docx]

**Advanced Stepwise Boosts High Conversion Efficiency in Intrinsic High Power-Density Mg₂Sn-GeTe Thermoelectric Generator**

Xinzhi Wu ^1, 3^, Longquan Wang ^1, 2, 3^, Airan Li ^1, 3^, Gang Wu ^1^, Zhao Hu^1, 2^, Fei Frank Yun ^1^, Takao Mori ^1, 2, *^

- 1. Research Center for Materials Nanoarchitectonics (MANA), National Institute for Materials Science (NIMS), Tsukuba, 305-0044, Japan
  2. Graduate School of Pure and Applied Science, University of Tsukuba, 1-1-1 Tennodai, Tsukuba, Ibaraki 305-8671, Japan
  3. Equally contributed

* Corresponding author, E-mail: mori.takao@nims.go.jp

**Debye-Callaway model**

Based on the Debye-Callaway model, the lattice thermal conductivity *κ*_L_ can be expressed as:

(1)

where *x*= ℏ*ω*/*κ_B_T* is the reduced phonon frequency, *ω* is the phonon frequency, *θ_D_* is the Debye temperature, *v* is the average phonon group velocity, and *τ* is the overall phonon scattering relaxation time. Phonon scattering mechanisms, including Umklapp (U) scattering, grain boundary (GB) scattering, and point defect (PD) scattering are considered in this work. Accordingly, *τ* can be expressed and simplified as:

$\tau^{-1}=\tau_{\text{PD}}^{-1}+\tau_{\text{U}}^{-1}+\tau_{\text{GB}}^{-1}=A\omega^{4}+B\frac{\omega^{2}T}{\nu^{3}}++\frac{\nu}{L}$ (2)

where $\tau_{\text{PD}}^{-1}$, $\tau_{\text{U}}^{-1}$, and$\tau_{\text{GB}}^{-1}$ are the phonon relaxation times for PD scattering, U scattering, and GB scattering, respectively. The fitting parameters *A*, *B*, and *L* are listed in Table S1.

Table S1. Parameters obtained by fitting the experimental lattice thermal conductivity of Mg_2_Sn_0.8_(Sb_0.5_Bi_0.5_)_0.2_ using the Debye-Callaway model.

| Materials | *A* | *B* | *L* |
| --- | --- | --- | --- |
| Mg_2_Sn_0.8_(Sb_0.5_Bi_0.5_)_0.2_ | 5.13E-42 | 2.20E-07 | 5.00E-07 |

**Calculation of Power and Conversion Efficiency**

In the thermoelectric modules tested by the commercial instrument Mini-PEM (Advance Riko), a temperature difference (Δ*T*) between the two ends induces a voltage (Δ*V*). The cold-side temperature was maintained at 293 K, while the hot-side temperatures varied between 373 K and 723 K. The Seebeck coefficient (*S*) is determined as the ratio of the generated voltage to the temperature difference. When an electric current (*I*) flows through the device, the current dependence of the power generation (*P*) can be described by the following equation:

$P=I\left(\text{Δ}V-IR\right)=RI^{2}+S\text{Δ}TI$ (3)

Where *R* denotes the internal resistance of the thermoelectric device. The maximum power generation (*P*_max_) can be evaluated from the current dependent power output. The heat flow (*Q*) through the thermoelectric device is defined by:

$Q=Q(0)+ST_{c}I+\frac{1}{2}I^{2}R$ (4)

Where *Q*(0) represents the heat contribution due to the temperature difference across the sample and additional thermal effects. Where *T*_c_ is the temperature on the low-temperature side. Finally, the conversion efficiency *η* is defined as the ratio of power output to the total heat flow:

$\eta=\frac{RI^{2}+S(T_{h}-T_{c})I}{-RI^{2}/2+ST_{h}I+Q(0)}$ (5)

The heat flow is evaluated by the flow calorimeter. In the flow calorimeter, the temperature difference between the inlet temperature in liquid (*T*_in_) and the outlet temperature in liquid (*T*_out_) and the velocity in liquid (*v*) depends on the heat flow in the sample. This relation is shown in the following equation.

$T_{out}-T_{in}=\frac{Q}{C}\frac{1}{\nu}$ (6)

The heat capacity per volume is shown in (*C*). The heat flow can be evaluated by the slope of the temperature difference between the inlet temperature in liquid the outlet temperature in liquid and the velocity in liquid.

**Figure S**

Figure S 1 The temperature-dependent TE properties of the Ge_0.9_Sb_0.1_Te bulk (a) Electrical resistivity, (b) Seebeck coefficient, (c) power factor, (d) thermal conductivity, (e) lattice thermal conductivity, and (f) *ZT.*

Figure S 2 Repeatable TE properties of Mg_2_Sn_0.8_(Sb_0.5_Bi_0.5_)_0.2_ TE materials. (a) Electrical resistivity, (b) Seebeck coefficient, (c) power factor, (d) thermal conductivity, and (e) *ZT.*

Figure S 3 XRD patterns of Mg_2_Sn-based TE materials

Figure S 4 Rietveld refinement analysis from the XRD pattern of Mg_2_Sn_0.8_(Sb_0.5_Bi_0.5_)_0.2_.

Figure S 5 Lattice constant (a) as a function of anion composition.


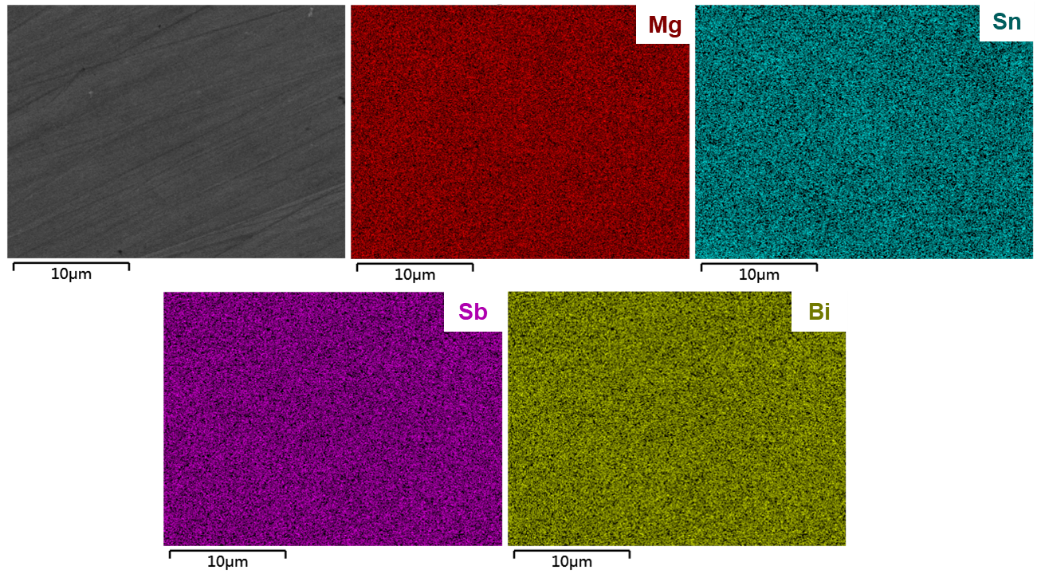


Figure S 6 SEM and EDS of Mg_2_Sn_0.8_(Sb_0.5_Bi_0.5_)_0.2_ TE materials.

Figure S 7 Pisarenko plot of Mg_2_Sn-based TE materials.

Figure S 8 Magnetic field (*B*) dependence of the Hall resistance (*V*_H_/*I*). The Hall coefficient (*R*_H_) is determined from the linear slope of the curve.

Figure S 9 A two-dimensional map correlating the *κ*_L_ with the sound velocity *ν.*

Figure S 10 Experimental and simulated *κ*_L_ of Mg_2_Sn_0.8_(Sb_0.5_Bi_0.5_)_0.2_ TE materials and comparison with the literature^[1]^.

Figure S 11 The TE quality factor *B* as a function of temperature.

Figure S 12 Ti/Ge_0.9_Sb_0.1_Te interface. (a) Resistance *R* versus position curve. (b) Magnified view of the interface region in (a), highlighting the interface resistance determined from the resistance jump across the probe displacement.


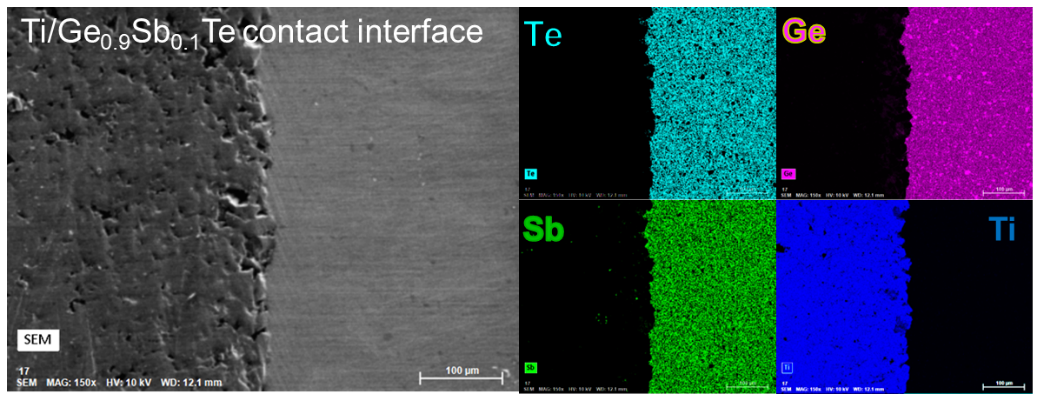


Figure S 13 Scanning electron microscopy (SEM) image and corresponding energy dispersive spectroscopy (EDS) elemental maps of the Ti/Ge_0.9_Sb_0.1_Te contact interface.

Figure S 14 Performance of Mg_2_Sn_0.8_(Sb_0.5_Bi_0.5_)_0.2_ single-leg TE device. (a) The Voltage 𝑉, (b) Output power 𝑃, (c) Heat flow at the cold side 𝑄𝑐, and (d) Conversion efficiency 𝜂 as a function of current 𝐼 under various temperature differences Δ𝑇 with photograph of the single leg setup in the commercial instrument Mini-PEM.

Figure S 15 Performance of Ge_0.9_Sb_0.1_Te single-leg TE device. (a) The Voltage 𝑉, (b) Output power 𝑃, (c) Heat flow at the cold side 𝑄𝑐, and (d) Conversion efficiency 𝜂 as a function of of current 𝐼 under various temperature differences Δ𝑇 with photograph of the single leg setup in the commercial instrument Mini-PEM.

Figure S 16 Comparison of experimental and predicted values of the power generation performance of the Mg_2_Sn_0.8_(Sb_0.5_Bi_0.5_)_0.2_-Ge_0.9_Sb_0.1_Te module. (a) Δ*T*-dependent 𝜔_max_, (b) Δ*T*-dependent 𝜂_max_.

Figure S 17 *P* with the cycle number, *T*_h_ cycling at 473–673 K.

**Reference**

[1] Y. B. Zhu, E. T. Dong, Z. J. Han, F. Jiang, J. H. Sui, W. Q. Zhang, W. S. Liu, Maximized atomic disordering approach boost the thermoelectric performance of Mg_2_Sn through the self-compensation effect and steric effect, *Acta Mater.* **2021**, *217*, 117172.
